# Influence of Surgery Preparation Time on Patient Outcomes

**DOI:** 10.1155/2024/6753210

**Published:** 2024-05-30

**Authors:** Hey-Jin Jang, Sun-Mi Lee

**Affiliations:** College of Nursing, The Catholic University of Korea, Seoul, Republic of Korea

## Abstract

**Aims:**

This study aimed to analyze the effects of the surgery preparation time on patient outcomes.

**Background:**

Postoperative complications have a decisive effect on postoperative survival. The anesthesia time is a crucial determinant of such complications. Competent operating room nurses can shorten the surgery preparation time, which is the time from when anesthesia is first administered to the making of the surgical incision. The shortening of this preparation time can shorten the anesthesia time and may reduce postoperative complications. However, discussion of this preparation time is insufficient. Therefore, this study analyzed the effect of the surgery preparation time on patient outcomes.

**Methods:**

From electronic health records data, this retrospective cohort study used the data of 1,944 patients who had been immediately admitted to the ICU after their surgery between 2017 and 2020. The patients were divided into two groups: ≥30 minutes preparation time and <30 minutes preparation time groups. We performed chi-squared tests and *t*-tests to determine differences in preoperation, intraoperation, and postoperation characteristics of the patients and patient outcomes based on the surgery preparation time. Furthermore, we performed a multiple logistic regression by including 12 adjusted variables to determine the influence of the surgery preparation time on patient outcomes.

**Results:**

Among the 1,944 patients, 820 were in the ≥30 minutes preparation time group and 1,124 in the <30 minutes preparation time group. The multiple logistic regression analysis showed that the surgery preparation time affects alertness (OR = 1.44; 95% CI: [1.09, 1.90]), ventilator application (OR = 1.32; 95% CI: [1.03, 1.70]), and length of stay in the ICU (OR = 1.69; 95% CI [1.16, 2.47]).

**Conclusions:**

The surgery preparation time affects postoperative patient outcomes. The competence of operating room nurses is the most essential aspect of the surgery preparation time. *Implications for Nursing Management*. It is important to analyze operating room nurses' tasks, standardize the tasks, and educate nurses according to their experience level to reduce the surgery preparation time and improve patient outcomes.

## 1. Background

The number of surgeries is increasing steeply every year worldwide [1]. While the risk of intraoperative complications is decreasing with the development of medical technologies, the risk of postoperative complications is increasing. Every year, tens of thousands of patients experience postoperative complications [2]. Notably, the risk of postoperative complications is higher in major surgeries than in minor ones, with an incidence rate of up to 25% [3].

Postoperative complications have been found to have a decisive effect on postoperative survival [4]. Reportedly, two million patients die every year due to postoperative complications [5]. In addition, the mortality rate within 60 days of surgery is 3.4 times higher if complications occur [6]. Common postoperative complications include pain, delirium, pressure ulcers, decreased bowel movement, infection, and bleeding. These complications result in patient outcomes such as admission to the intensive care units (ICUs), prolonged hospitalization, and increased healthcare costs [7–9].

The factors affecting postoperative patient outcomes can be delineated as internal and external. Internal factors are factors related to the patient, such as demographic characteristics, lifestyle, diseases, surgical history, degree of activity, and intraoperative status [10, 11]. External factors are related to the surgical processes, such as the time and method of anesthesia, anesthetic agent, surgery time, surgery site and method, surgery position, and intraoperative specifics [11–13].

Among these factors, the factors that are controllable and can reduce postoperative complications and improve patient outcomes are largely external. Notably, among all external factors, anesthesia time has the most significant influence. Prolonged anesthesia time is associated with the risk of nausea, vomiting, infection, and bleeding as well as venous thromboembolism in serious cases [14]. It contributes to increased length of stay and healthcare costs, as do postoperative complications [15–17].

The anesthesia time can be divided into three periods: the surgery preparation, surgery, and dressing periods. The “surgery preparation period” begins with the initiation of anesthesia and continues until the surgical incision is made [18–21]. During this period, the patient is prepared for surgical positioning [19, 22, 23], while operating room (OR) nurses endeavor to maintain a sterile environment by handling surgical equipment, ensuring cleanliness, and performing sterilization. They also assist the patient in donning the surgical attire and maintaining its sterility. These efforts are aimed at preventing surgical site infections [23–26]. The “surgery time” starts when the surgical incision is made and ends when sutures are made [22, 27, 28]. This is the duration of the surgical procedure and is recorded as the total time on the surgical record sheet. Reducing this duration can positively affect patient outcomes [8, 29, 30]. Finally the “dressing period” spans from the completion of the operation to the time the anesthesia completely ends. During this period, the patient's condition is checked and necessary disinfection and cleanup actions are performed. This period includes the time taken for the patient to prepare for movement in the operating room.

The surgery preparation time is when the work of OR nurses is the most concentrated. Nursing work performed during the surgery preparation time can be divided into the tasks performed by scrub and circulatory nurses in sterile and nonsterile fields, respectively. Scrub nurses are tasked with various responsibilities aimed at facilitating the surgical procedure. These responsibilities encompass delineating the parameters of the sterile field, arranging sterilized surgical instruments, and aiding the surgical team in donning gloves and gowns [31, 32].

Meanwhile, circulatory nurses primarily handle tasks outside the sterile field in the OR. This includes protecting the integrity of the sterile field, assisting the scrub nurse in the gowning of the surgical team, delivering the items needed for surgery into the sterile field, and arranging the positioning of surgical equipment [25, 26]. Thus, the surgery preparation period is a critical juncture in which OR nurses' expertise and proficiency exert a significant influence on patient safety and surgical efficiency.

Competent OR nurses can shorten the surgery preparation period, which can shorten the anesthesia time, thereby contributing to reducing the instances of postoperative complications. Most studies have analyzed OR nursing tasks and quantitatively measured the time required to prepare for surgery. Consequently, research remains scant on the association between the surgery preparation time and patient outcomes [18, 19, 21]. Thus, this study aimed to analyze the impact of surgery preparation time on postoperative patient outcomes, such as the presence or absence of medical interventions.

## 2. Methods

### 2.1. Study Design

This was a retrospective cohort study conducted using electronic health record (EHR) data.

### 2.2. Data Source

This study used an EHR dataset that was created for the primary study to predict pressure injuries in ICUs. This EHR dataset was extracted from the EHR system of the University Hospital in Seoul, South Korea. It contained the clinical data of 6,555 patients who were hospitalized and discharged from medical and surgical ICUs between January 1, 2017, and February 28, 2020. The dataset contained data on 1,106 variables extracted from various sources, such as anesthesia, surgery, and recovery records, nursing information records, clinical observation records, laboratory clinical pathology tests, medication administration records, blood transfusion records, instrument and device insertion and removal records, and nursing diagnoses.

### 2.3. Study Population

**Figure 1:**
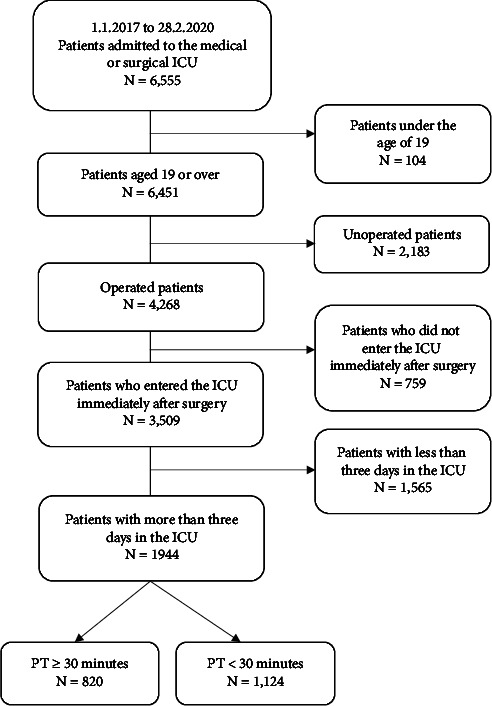
Flowchart of the selection of the study population.

Of the 6,555 individuals enrolled in the primary study, we used the data of 1,944 individuals admitted to the ICU immediately after their surgery (Figure 1). The data of patients who were aged 19 or older and stayed in the ICU for three days or more were included in our analysis. We excluded the data of patients who were admitted to the general ward after surgery and were later transferred to the ICU due to increasing severity.

### 2.4. Study Variables

From the data source, we used the data on surgery preparation time (the main independent variable), eight patient outcome variables, patient demographic variables, and adjusted variables in our analysis.

#### 2.4.1. Surgery Preparation Time

Owing to a lack of a standard surgery preparation time, criteria for surgery preparation time vary across studies. In this study, the surgery preparation time was considered to be the period between the initiation of anesthesia to the making of the surgical incision. According to previous research, the average surgery preparation time ranges from 10 minutes to 57 minutes [18–21]. In this study, which targeted 1,944 participants, the average surgical preparation time was 30 minutes. Consequently, the study population was classified based on whether their surgery preparation time was 30 minutes or longer (≥30 minutes preparation time group) or less than 30 minutes (<30 minutes preparation time group).

#### 2.4.2. Patient Outcomes

To analyze the impact of the surgery preparation time on patient outcomes, we selected the postoperative patient outcomes, primarily nursing-sensitive ones, that could be observed in the ICU postoperatively. Consequently, eight outcomes were selected: alertness, number of nursing diagnoses related to postoperative complications, respiratory nursing needs score, ventilator application, restraint application, transfusion, use of narcotics, and ICU length of stay (ICU LOS).

The recovery period of surgery patients is classified into three stages: 24 hours to within 7 days, 28 to 60 days, and 6 weeks to 3 months [33]. The first stage of recovery (24 hours to within 7 days) is the acute postoperative phase and a crucial period in postoperative management. Since we speculated that the impact of surgery preparation time would lessen over time after the seven-day mark, we selected postoperative patient outcomes occurring within seven days postoperation as the outcome variables [33].

Alertness was determined based on the percentage of times the patient was found to be alert in the level of consciousness assessment (alert, confusion, drowsy, stuporous, semicoma, and coma) within seven days of being admitted to the ICU. This percentage was categorized as ≥ 50% or <50%. The number of nursing diagnoses related to postoperative complications was determined based on the daily average number of diagnoses over seven days of being admitted to the ICU. We selected and analyzed 60 nursing diagnoses related to postoperative complications, such as “impaired gas exchange,” “electrolyte imbalance,” and “risk of deficient fluid volume,” from the diagnoses in use at the time at the target hospital. The daily average number of nursing diagnoses related to postoperative complications per individual was found to be 5.75. Therefore, we categorized the study population as individuals with >5.75 diagnoses related to postoperative complications and those with ≤5.75 diagnoses.

The respiratory nursing needs score is the score of respiratory care needs that is measured daily on a scale from 0 to 82. The higher the score, the more respiratory care needs are evident. In this study, the study population was categorized as individuals with a score of ≤2 and those with a score of >2. The reference score of 2 indicated basic respiratory care needs (such as deep breathing and assisted coughing and the use of a spirometer). Scores greater than two suggest that additional interventions are needed, such as oxygen therapy, suction, or a ventilator.

Regarding ventilator and restraint application, we categorized the study population based on whether they were applied within seven days of being admitted to the ICU. Similarly for transfusion, we categorized the study population based on whether a transfusion was performed within seven days of being admitted to the ICU. The use of narcotics was determined based on whether the narcotic analgesics listed in the medication records were administered within seven days of ICU admission. The ICU LOS was defined as the number of days spanning from ICU admission to discharge. The study population was categorized as individuals who stayed in the ICU for ≤7 days and those who stayed for >7 days.

#### 2.4.3. Adjusted Outcomes

From the preoperational, intraoperational, and postoperational factors in the data source, we selected 12 adjusted variables: the body mass index (BMI), score on the American Society of Anesthesiologists Physical Status Classification System (ASA Physical Status Classification System), surgery method, systolic blood pressure (SBP), pulse, surgery time, total recovery time, score on the Acute Physiology and Chronic Health Evaluation (APACHE II), blood urea nitrogen (BUN), creatinine, creatinine phosphokinase (CPK), and lactate dehydrogenase (LDH).

The ASA score evaluates a patient's physical condition before surgery. This score was categorized as normal (I), mild systemic disease (II), severe systemic disease (III), severe systemic disease having a constant threat to life (IV), not expected to survive without the operation (V), or brain-dead patient for donation (VI).

The surgery method was categorized as surgery with scope, surgery without scope, or undefined based on the name of the operation. We classified the surgery method based on the use of a scope because using a scope has a grave impact on the surgical approach as well as the work environment of OR nurses [20, 34]. More specifically, scope-assisted surgeries require specific equipment, such as endoscopic instruments (SCOPE), CO2, intraabdominal illumination (light cable), and specially modified instruments [34, 35]. The surgery preparation time varies owing to the process of preparing these items.

The SBP and pulse were determined based on the average SBP and pulse during surgery, respectively. The surgery time was calculated as the time starting from the time the incision was made to the time the incision was closed. The total recovery time was defined as the time from PACU admission to transfer to the patient room.

The APACHE II score indicates the severity of the disease within 24 hours of admission to the ICU. The score is calculated based on 12 items (body temperature, mean arterial pressure, blood pH, heart rate, respiratory rate, PaO2, serum sodium, serum potassium, creatinine, hematocrit, white blood cell count, and Glasgow Coma Scale) and ranges from 0 to 71. Higher scores indicate heightened severity. Finally, among the initial blood tests conducted upon admission to the ICU after surgery, statistically significant results were observed for BUN, creatinine, CPK, and LDH.

### 2.5. Data Analysis

The study population was divided into two groups: the group with a surgery preparation time of 30 minutes or longer and the group with a surgery preparation time of less than 30 minutes. Chi-squared tests were performed on categorical variables, while continuous variables were analyzed using a *t*-test. Then, we performed a multiple logistic regression analysis by including the 12 adjusted variables to identify the impact of the surgery preparation time on the eight patient outcomes. These statistical analyses were performed using SAS version 9.4.

### 2.6. Ethical Considerations

This study was conducted after obtaining approval from the research institution's Data Utilization Review Board and Institutional Review Board (KC23RISI0254). Personally identifiable data were deleted and replaced with numbers for safe management of the data used in this study.

## 3. Results

### 3.1. Preoperation, Intraoperation, and Postoperation Characteristics of the Study Population

Table 1 presents the results of analyzing preoperation, intraoperation, and postoperation characteristics of the study population based on the surgery preparation time. Among the study population, 820 individuals had a surgery preparation time of ≥30 minutes, while 1,124 had a surgery preparation time of <30 minutes. The mean preparation time was 45.08 minutes in the ≥30 minutes preparation time group and 18.45 minutes in the <30 minutes preparation time group.

The age and sex of the study population did not differ significantly between the two groups of the surgery preparation time (*p* > 0.05). In the preoperative period, the proportion of obese patients (those with a BMI of >23 kg/m^2^) was higher in the ≥30 minutes preparation time group (48.05%) than in the <30 minutes preparation time group (39.15%; *p* < 0.001). Furthermore, the proportion of patients with ASA classifications II and III was higher in the ≥30 minutes preparation time group (54.76%; *p* < 0.001). Meanwhile, the proportion of patients with ASA classifications IV and V was higher in the <30 minutes preparation time group (51.51%; *p* < 0.01), indicating a comparatively higher degree of severity.

During the intraoperative period, the proportion of patients who underwent surgery with a scope was higher in the ≥30 minutes preparation time group (16.10%; *p* < 0.01), while patients who underwent surgery without scope were more in number in the <30 minutes preparation time group (69.13%; *p* < 0.01). The proportion of patients with an SBP of ≥120 mmHg (39.77%; *p* > 0.001) and those with a pulse rate of ≥100/minute (13.70%; *p* < 0.01) was higher in the <30 minutes preparation time group. However, the surgery time (285.10 ± 149.22 min) was longer in the group with ≥30 minutes of preparation time (*p* < 0.001).

In the postoperative period, the recovery time (77.05 ± 32.59 min) was longer in the group with more than 30 minutes of preparation time (*p* < 0.05). In addition, levels of BUN (34.95 ± 28.05 mg/dL) and creatinine (2.99 ± 3.21 mg/dL) were higher in the group with less than 30 minutes of preparation time (*p* < 0.001 for both). However, CPK levels (239.07 ± 282.45 U/L) and LDH levels (725.10 ± 536.85 U/L) tended to be significantly higher in the group with more than 30 minutes of preparation time (*p* < 0.001 for both) (Table 1).

### 3.2. Influence of the Surgery Preparation Time on Postoperative Patient Outcomes

Table 2 presents the results of analyzing the eight postoperative patient outcomes based on the surgery preparation time. Five of the eight outcomes differed significantly based on the surgery preparation time. The ≥30 minutes preparation time group had a higher proportion of patients with less than 50% alertness (29.02%; *p* < 0.05), those with a score of >2 for respiratory nursing needs (36.22%; *p* < 0.05), those who had undergone a transfusion (56.59%; *p* < 0.01), those who had been administered narcotics (62.68%; *p* < 0.001), and those who stayed in the ICU for >7 days (13.90%; *p* < 0.001).

Table 3 presents the results of the multiple logistic regression analysis. The surgery preparation time had a statistically significant effect on alertness, ventilator application, and ICU LOS. The ≥30 minutes preparation time group was at high risk for less than 50% alertness (OR = 1.44; 95% CI: [1.09, 1.90]) and ventilator application within seven days of being admitted to the ICU (OR = 1.32; 95% CI: [1.03, 1.70]). It was also at a high risk of staying in the ICU for more than seven days (OR = 1.69; 95% CI: [1.16, 2.47]).

## 4. Discussion

We included 12 adjusted variables in our analysis and found that a longer surgery preparation time lowers the rate of alertness within seven days of the operation and increases the likelihood of ventilator application and extension of the ICU LOS.

The significant decrease in the level of consciousness with an increase in the surgery preparation time is presumed to be due to the accompanying increase in the anesthesia time. General anesthesia impacts the nervous system [36] and, consequently, the level of consciousness. Therefore, shortening the surgery preparation time (and, in turn, shortening the overall anesthesia time) can affect the patient's level of consciousness after surgery. Improving the capacity of OR nurses can help shorten the surgery preparation time and improve patients' level of consciousness after surgery.

Studies have found that the “BMI, BUN, creatinine, surgery time, and surgery method, and scores on the ASA and APACHE II,” prolong ICU LOS [37–42]. However, in this study, the surgery preparation time increased ICU LOS even after adjusting for these variables. Regarding surgery or medical treatment, studies have reported that the determinants of ICU LOS are primarily patients' intrinsic factors or factors on which nurses' influence is weak. This study removed the intrinsic and medical factors and confirmed that the surgery preparation time (a factor on which OR nurses' influence is the most concentrated) affects ICU LOS. Considering that the competence of OR nurses can reduce the surgery preparation time, positive patient outcomes can be expected if their competency is enhanced and thus efficient preparation time management becomes possible.

After adjusting for factors related to the patient's condition, we found that there is a higher risk of ventilator application when the surgery preparation time exceeds 30 minutes. In other words, when patient condition and surgical-related variables are similar, patients with longer surgical preparation times are at a higher risk of requiring mechanical ventilation. this finding suggests that the length of the surgery preparation time affects the occurrence of ventilator application.

Previous studies have highlighted BMI and surgery time as key factors influencing ventilator application [43–47]. However, this study revealed that the surgery preparation time, which can be controlled by OR nurses, may influence ventilator application. This finding contributes to the research on OR nursing and patient outcomes and emphasizes the importance and influence of OR nursing [48]. It also confirms the importance of OR nurses in the surgical process. From this perspective, further research on the surgery preparation time is imperative for enhancing surgical patient outcomes.

In the univariate analysis, respiratory nursing needs, transfusion, and use of narcotics differed significantly based on the surgery preparation time. However, these factors became statistically insignificant in the multiple logistic regression analysis. This result indicates that factors that have been found to influence respiratory nursing needs, transfusion, and use of narcotics in previous studies, such as BMI, score on the ASA, surgery time, and type of surgery, may act in combination [49–56]. Patients with higher scores on the ASA exhibit greater respiratory nursing needs [49]. Furthermore, as the surgery duration increases, the volume of bleeding typically increases, thereby raising the likelihood of requiring transfusions [57, 58]. This, in turn, may lead to the increased use of narcotic analgesics to manage pain. Consequently, it can be inferred that factors related to patient condition and surgical parameters exert a more significant influence on elevating the respiratory nursing needs score and the risks associated with transfusions and narcotics use, rather than the surgery preparation time. Further research is warranted on the surgery preparation time to enhance surgical outcomes for patients.

The number of nursing diagnoses related to postoperative complications did not differ significantly based on the surgery preparation time. Previous studies have indicated that patient factors influence nursing diagnoses [49]. However, no significant differences were observed in this study both before and after adjustment. This could be attributed to the fact that this study examined postoperative patient outcomes occurring within seven days of being admitted to the ICU [33]. Considering that the early stages of postsurgery are centered on preventing complications, the number of nursing diagnoses related to postoperative complications may have remained relatively constant or uniform during the 7-day period.

Finally, the surgery preparation time had no statistically significant impact on the use of physical restraint. Physical restraint is used to keep the treatment instruments in place and prevent injuries from falls or self-removal of medical devices [59]. It is recommended not to restrain patients physically, but the ICU seems to be an exception [59, 60]. It can be speculated that physical restraint was used in most cases in this study, considering that this study targeted ICU patients [61–63] and the higher likelihood of its use among patients on ventilator support.

This study investigated the impact of reducing the surgery preparation time (and, consequently, the anesthesia time) on patient outcomes. Some studies have suggested methods to reduce the surgery preparation time by focusing on the arrival time of the surgeon to the OR [18]. The surgeon's timely arrival to the OR shortens the endotracheal tube repositioning time and ensures a quicker process of confirming the position of equipment and devices. However, other studies have argued otherwise. One study reported that the surgeon's OR arrival time does not have a significant impact on the surgery preparation time [64]. Due to this discrepancy in the findings, we could not establish clear evidence of the shortening of the surgery preparation time in relation to the surgeon's OR arrival time.

Many studies have stated that the surgery preparation time is a source of inefficiency and an aspect that needs improvement in the OR workflow, regardless of the surgical site [65–67]. However, technological advances are transforming the OR environment and surgery methods [18, 34, 68]. Now, even the same surgery method requires highly specialized competencies due to the various surgery methods used by each surgeon and department [22, 24, 65, 69–71]. The time that connects these varied surgical environments and surgery methods is the surgery preparation time. That is, the surgery preparation time depends on the competency of the OR nurses who adapt to the changes in the OR environment and understand the various surgery methods [18].

OR nurses are unique and irreplaceable professionals in healthcare. They are responsible for guaranteeing intraoperative safety and managing and controlling intraoperative asepsis, instrument use, infection and complications, biological samples, and the technology to be employed in the OR [32]. However, the rapid changes in surgery methods and equipment increase the surgery preparation time [20]. As the surgery preparation time extends, tension intensifies during this period, resulting in an escalated workload at this stage [72]. Consequently, it makes the tasks of OR nurses more complex. Despite this, OR nursing is relatively undervalued, and the appropriate nursing cost is yet to be established [73]. Therefore, further research is needed on the surgery preparation time and specific tasks of OR nurses during this period.

## 5. Implications and Limitations

This study has important implications for nursing management, as it examined the relationship between postoperative patient outcomes and the surgery preparation time, a period during which OR nurses play a crucial role. The findings emphasize the significance of the surgery preparation time and the need to raise awareness about the importance of OR nursing. Based on the results, we propose several policy recommendations for OR nursing management.

First, it is crucial to conduct a precise analysis of the tasks performed during the surgery preparation time, a period when most OR nursing tasks are performed. Despite advancements in surgical techniques and methods and the surgical environment, there remains a lack of discussion on the surgery preparation time. This is evident in the continued reliance on two OR nurses, regardless of changes in the surgical environment and methods, without considering variations in surgery types or complexities. Although tasks during the surgery preparation time may seem routine, they involve various elements based on theoretical knowledge and principles. This situation indicates that despite an increase in the workload of OR nurses compared to that in the past, the current distribution system fails to reflect these changes adequately. Therefore, there is a need to reassess and adjust the work structure and processes of OR nurses. This will provide a foundation for reducing the surgery preparation time.

Second, a standardized framework must be developed for the surgery preparation time. A standardized process must be created for the surgery preparation time based on factors such as the surgical site, technique, and position to minimize errors and enhance efficiency, which would ultimately reduce the preparation time and help save costs.

Third, the OR is one of the most specialized areas within a hospital. Therefore, a lack of sufficient understanding of and proficiency in the preoperative preparation process can significantly reduce work efficiency [74–76]. Thus, OR nurses' qualifications should be redefined in terms of knowledge, skills, attitude, and experiences, and education programs must be developed to address individual educational needs [64, 76, 77]. Finally, it is essential to move away from the traditional nursing education framework that focuses on “preoperative nursing” and “postoperative nursing.” Instead, it is important to define “intraoperative nursing” and widely communicate its importance to provide a new perspective on the role of OR nurses.

This study has some limitations. First, we did not include environmental and structural factors of ORs and the ICU because they were not included in the data source. Second, an intrinsic bias must be acknowledged, as this study was a retrospective data analysis of a single institution. Finally, the generalizability of our results is limited because the study population was limited. Nonetheless, this study offers a new perspective on the clinical aspect of surgical nursing education, serving as a turning point in explaining the importance of “quantitative” nursing beyond the existing “qualitative” nursing perspective. Such an approach aims to foster a deeper understanding of the role and education of OR nurses, emphasizing the impact of nursing during surgery on patient recovery and treatment processes. The findings of this study are expected to provide important implications for the education and practice of OR nurses.

## 6. Conclusion

This study shows that a surgery preparation time exceeding 30 minutes reduces the rate of alertness, prolongs the patient's stay in the ICU, and increases the likelihood of ventilator application. Thus, the surgery preparation time should be estimated based on the surgical environment and methods from the moment a surgical schedule is confirmed to improve patient outcomes. In addition, efforts should be made to establish an efficient and reasonable management system for environmental, structural, and workforce issues to ensure that the surgery preparation time is handled efficiently according to the surgery method.

## Figures and Tables

**Table 1:** Preoperation, intraoperation, and postoperation patient characteristics (*N* = 1,944).

Variable	Categories		Surgery preparation time		*χ* ^2^/*t*	*P* value
			≥30 minutes (*n* = 820)	<30 minutes (*n* = 1,124)		
			*n* (%) or mean ± SD			

*Surgery preparation time (minutes)*			45.08 ± 17.07	18.45 ± 6.16	−42.70	<0.001
*Preoperation*						
	Age		59.80 ± 15.24	59.46 ± 16.04	−0.47	0.64
	Sex	Male	510 (62.20)	692 (61.57)	0.08	0.78
		Female	310 (37.80)	432 (38.43)		
	BMI (kg/m^2^)	<18.5	49 (5.98)	73 (6.49)	15.52	<0.001
		18.5–23	377 (45.98)	611 (54.36)		
		>23	394 (48.05)	440 (39.15)		
	ASA	I	71 (8.66)	79 (7.03)	42.81	<0.001
		II, III	449 (54.76)	466 (41.46)		
		IV, V	300 (36.59)	579 (51.51)		
*Intraoperation*						
	Surgery method	With scope	132 (16.10)	134 (11.92)	12.15	0.002
		Without scope	507 (61.83)	777 (69.13)		
		Undefined	181 (22.07)	214 (18.95)		
	SBP (mmHg)	<120	586 (71.46)	677 (60.23)	26.28	<0.001
		≥120	234 (28.54)	447 (39.77)		
	DBP (mmHg)	<60	188 (22.93)	228 (20.28)	1.97	
		≥60	632 (77.07)	896 (79.72)		
	Pulse (bpm)	<100	740 (90.24)	970 (86.30)	6.97	0.04
		≥100	80 (9.76)	154 (13.70)		
	Surgery time (minutes)		285.10 ± 149.22	207.94 ± 139.51	−11.57	<0.001
	Dressing time (minutes)		8.70 ± 3.99	8.47 ± 3.51	−1.30	0.19
*Postoperation*						
	Total recovery time (minutes)		77.05 ± 32.59	74.03 ± 30.13	−2.07	0.06
	APACHE II		12.50 ± 5.49	14.53 ± 6.11	7.67	<0.001
	BUN		22.24 ± 20.95	34.95 ± 28.05	11.44	<0.001
	Creatinine		1.53 ± 1.97	2.99 ± 3.21	12.33	<0.001
	CPK		239.07 ± 282.45	169.33 ± 233.16	−5.78	<0.001
	LDH		725.10 ± 536.85	636.56 ± 434.80	−3.88	<0.001

SD, standard deviation; BMI, body mass index; ASA physical status classification system (I, a normal healthy patient; II, a patient with mild systemic disease; III, a patient with severe systemic disease; IV, a patient with severe systemic disease that is a constant threat to life; V, a moribund patient who is not expected to survive without the operation); SBP, systolic blood pressure; DBP, diastolic blood pressure; surgery time, time from incision to close; dressing time, time from close to the end of anesthesia; APACHE II score, acute physiology and chronic health evaluation; BUN, blood urea nitrogen; CPK, creatinine phosphokinase; LDH, lactate dehydrogenase.

**Table 2:** Results of analyzing postoperative patient outcomes based on the surgery preparation time (*N* = 1,944).

Variable	Categories	≥30 minutes (*n* = 820)	<30 minutes (*n* = 1,124)	*χ* ^2^/*t*	*P* value
		*n* (%) or mean ± SD			
Alertness	<50%	238 (29.02)	381 (33.90)	5.19	0.02
	≥50%	582 (70.98)	743 (66.10)		

ICU LOS(days)	>7	114 (13.90)	77 (6.85)	26.61	<0.001
	≤7	706 (86.10)	1047 (93.15)		

Ventilator application	Yes	620 (75.61)	884 (78.65)	2.5	0.11
	No	200 (24.39)	240 (21.35)		

Respiratory nursing needs score (daily average)	>2	297 (36.22)	353 (31.41)	4.94	0.03
	≤2	523 (63.78)	771 (68.59)		

Transfusion	Yes	464 (56.59)	563 (50.09)	8.03	0.004
	No	356 (43.41)	561 (49.91)		

Use of narcotics	Yes	514 (62.68)	594 (52.85)	18.71	<0.001
	No	306 (37.32)	530 (47.15)		

Number of nursing diagnoses related to postoperative complications (daily average)	>5.75	402 (49.02)	557 (49.56)	0.05	0.82
	≤5.75	418 (50.98)	567 (50.44)		

Restraint application	Yes	204 (24.88)	230 (20.46)	2.19	0.14
	No	616 (75.12)	894 (79.54)		

SD, standard deviation; ICU, intensive care unit.

**Table 3:** Results of determining the influence of the surgery preparation time on postoperative patient outcomes (*N* = 1,944).

Variable	Category	Β	OR (CI)
Alertness	<50%	0.1829	1.44 (1.09–1.90)
ICU LOS (days)	>7	0.2627	1.69 (1.16–2.47)
Ventilator application	Yes	0.1393	1.32 (1.03–1.70)
Respiratory nursing needs score (daily average)	>2	−0.0919	0.83 (0.67–1.04)
Transfusion	Yes	−0.0039	0.99 (0.80–1.23)
Use of narcotics	Yes	−0.0341	0.93 (0.74–1.20)
Number of nursing diagnoses related to postoperative complications (daily average)	>5.75	−0.0785	0.86 (0.70–1.05)
Restraint application	Yes	0.1052	1.23 (0.95–1.61)

ICU: intensive care unit; LOS: length of stay; adjusted variables: BMI (Body Mass Index), ASA (physical status classification system), surgery method, SBP (systolic blood pressure), pulse, surgery time, total recovery time, APACHE II (Acute Physiology and Chronic Eealth evaluation) score, BUN (blood urea nitrogen), creatinine, CPK (creatinine phosphokinase), and LDH (lactate dehydrogenase).

## Data Availability

The data utilized in this paper are not available for sharing due to the inclusion of patient personal information and privacy or ethical restrictions.

## References

[1] Weiser T. G., Haynes A. B., Molina G. (2015). Estimate of the global volume of surgery in 2012: an assessment supporting improved health outcomes. *The Lancet*.

[2] The International Surgical Outcomes Study group (2016). Global patient outcomes after elective surgery: prospective cohort study in 27 low-, middle- and high-income countries. *British Journal of Anaesthesia*.

[3] World Health Organization (2022). Safe surgery. https://www.who.int/teams/integrated-health-services/patient-safety/research/safe-surgery.

[4] Pearse R. M., Harrison D. A., James P. (2006). Identification and characterisation of the high-risk surgical population in the United Kingdom. *Critical Care*.

[5] Weiser T. G., Regenbogen S. E., Thompson K. D. (2008). An estimation of the global volume of surgery: a modelling strategy based on available data. *The Lancet*.

[6] Khuri S. F., Henderson W. G., DePalma R. G., Mosca C., Healey N. A., Kumbhani D. J. (2005). Determinants of long-term survival after major surgery and the adverse effect of postoperative complications. *Annals of Surgery*.

[7] Hsieh C. E., Hsu Y. L., Lin K. H. (2021). Association between surgical volumes and hospital mortality in patients: a living donor liver transplantation single center experience. *BMC Gastroenterology*.

[8] Cheng H., Chen B. P., Soleas I. M., Ferko N. C., Cameron C. G., Hinoul P. (2017). Prolonged operative duration increases risk of surgical site infections: a systematic review. *Surgical Infections*.

[9] Tevis S. E., Kennedy G. D. (2013). Postoperative complications and implications on patient-centered outcomes. *Journal of Surgical Research*.

[10] Jin T., Jin Y., Lee S. M. (2021). Medication use and risk of delirium in mechanically ventilated patients. *Clinical Nursing Research*.

[11] Zhang W. Y., Wu W. L., Gu J. J. (2015). Risk factors for postoperative delirium in patients after coronary artery bypass grafting: a prospective cohort study. *Journal of Critical Care*.

[12] An Y. S., Jin Y., Jin T., Hur E. Y., Lee S. M. (2019). Operative and anaesthetic factors influencing on delirium in the intensive care unit: an analysis of electronic health records. *Journal of Clinical Nursing*.

[13] Winter A., Steurer M. P., Dullenkopf A. (2015). Postoperative delirium assessed by post anesthesia care unit staff utilizing the Nursing Delirium Screening Scale: a prospective observational study of 1000 patients in a single Swiss institution. *BMC Anesthesiology*.

[14] Mlodinow A. S., Khavanin N., Ver Halen J. P., Rambachan A., Gutowski K. A., Kim J. Y. (2015). Increased anaesthesia duration increases venous thromboembolism risk in plastic surgery: a 6-year analysis of over 19,000 cases using the NSQIP dataset. *Journal of Plastic Surgery and Hand Surgery*.

[15] Phan K., Kim J. S., Kim J. H. (2017). Anesthesia duration as an independent risk factor for early postoperative complications in adults undergoing elective ACDF. *Global Spine Journal*.

[16] Phillips B. T., Wang E. D., Rodman A. J. (2012). Anesthesia duration as a marker for surgical complications in office-based plastic surgery. *Annals of Plastic Surgery*.

[17] Eappen S., Flanagan H., Lithman R., Bhattacharyya N. (2007). The addition of a regional block team to the orthopedic operating rooms does not improve anesthesia-controlled times and turnover time in the setting of long turnover times. *Journal of Clinical Anesthesia*.

[18] Mazzei W. J. (1994). Operating room start times and turnover times in a university hospital. *Journal of Clinical Anesthesia*.

[19] Ranganathan P., Khanapurkar P., Divatia J. V. (2013). Utilization of operating room time in a cancer hospital. *Journal of Postgraduate Medicine*.

[20] Sasano N., Morita M., Sugiura T., Sasano H., Tsuda T., Katsuya H. (2009). Time progression from the patient’s operating room entrance to incision: factors affecting anesthetic setup and surgical preparation times. *Journal of Anesthesia*.

[21] Costa Jr A. d. S., Leão L. E. V., Novais M. A. P. D., Zucchi P. (2015). An assessment of the quality indicators of operative and non-operative times in a public university hospital. *Einstein (Sao Paulo)*.

[22] Ashraf M., Kamboh U. A., Raza M. A. (2022). Prospective elective neurosurgical theater utilization audit in Pakistan: problems in a public tertiary care hospital and proposed solutions from lower-middle-income country. *Asian Journal of Neurosurgery*.

[23] Harders M., Malangoni M. A., Weight S., Sidhu T. (2006). Improving operating room efficiency through process redesign. *Surgery*.

[24] Clark A., Dackiw A. P., White W. D. (2016). Early endocrine attending surgeon presence increases operating room efficiency. *Journal of Surgical Research*.

[25] Pasquer A., Ducarroz S., Lifante J. C., Skinner S., Poncet G., Duclos A. (2024). Operating room organization and surgical performance: a systematic review. *Patient Safety in Surgery*.

[26] Jenkins D. R. (2017). Nosocomial infections and infection control. *Medicine*.

[27] Dorjey Y., Tshomo Y., Wangchuk D. (2023). Evaluation of decision to delivery interval and its effect on feto-maternal outcomes in Category-I emergency cesarean section deliveries in Phuentsholing General Hospital, 2020: a retrospective cross-sectional study. *Health Science Reports*.

[28] Stahl J. E., Egan M. T., Goldman J. M. (2005). Introducing new technology into the operating room: measuring the impact on job performance and satisfaction. *Surgery*.

[29] Wang Q., Goswami K., Shohat N., Aalirezaie A., Manrique J., Parvizi J. (2019). Longer operative time results in a higher rate of subsequent periprosthetic joint infection in patients undergoing primary joint arthroplasty. *The Journal of Arthroplasty*.

[30] Scigliano N. M., Carender C. N., Glass N. A., Deberg J., Bedard N. A. (2022). Operative time and risk of surgical site infection and periprosthetic joint infection: a systematic review and meta-analysis. *The Iowa Orthopaedic Journal*.

[31] Alayemi J., Ten Ham-Baloyi W., Jardien-Baboo S. (2024). Nurses’ knowledge regarding recommended practices on using surgical attire in operating theatre. *Health SA Gesondheid*.

[32] von Vogelsang A. C., Swenne C. L., Gustafsson B. A., Falk Brynhildsen K. (2020). Operating theatre nurse specialist competence to ensure patient safety in the operating theatre: a discursive paper. *Nursing Open*.

[33] Bowyer A. J., Royse C. F. (2016). Postoperative recovery and outcomes–what are we measuring and for whom?. *Anaesthesia*.

[34] Spaner S. J., Warnock G. L. (1997). A brief history of endoscopy, laparoscopy, and laparoscopic surgery. *Journal of Laparoendoscopic & Advanced Surgical Techniques*.

[35] Zheng B., Fung E., Fu B., Panton N. M., Swanström L. L. (2015). Surgical team composition differs between laparoscopic and open procedures. *Surgical Endoscopy*.

[36] Vutskits L., Xie Z. (2016). Lasting impact of general anaesthesia on the brain: mechanisms and relevance. *Nature Reviews Neuroscience*.

[37] Niewińsk G., Raszeja-Wyszomirska J., Główczyńska R. (2018). Risk factors of prolonged ICU stay in liver transplant recipients in a single-center experience. *Transplantation Proceedings*.

[38] Tribuddharat S., Sathitkarnmanee T., Ngamsaengsirisup K., Wongbuddha C. (2018). Validation of Open-Heart Intraoperative Risk score to predict a prolonged intensive care unit stay for adult patients undergoing cardiac surgery with cardiopulmonary bypass. *Therapeutics and Clinical Risk Management*.

[39] Toptas M., Sengul Samanci N., Akkoc İ. (2018). Factors affecting the length of stay in the intensive care unit: our clinical experience. *BioMed Research International*.

[40] Sveshnikova N. S., Paleev F. P. (2016). Factors affecting duration of stay in IntensiveCare Unit after coronary artery bypass surgery. *Kardiologiia*.

[41] Wallner J., Schwaiger M., Edmondson S. J. (2021). Effects of pre-operative risk factors on intensive care unit length of stay (ICU-LOS) in major oral and maxillofacial cancer surgery. *Cancers*.

[42] Ogilvie J. W., Wilkes A. W., Hobbs D. J., Smith J. R., Dull M. B., Luchtefeld M. A. (2020). The effect of chronic preoperative opioid use on surgical site infections, length of stay, and readmissions. *Diseases of the Colon & Rectum*.

[43] De Jong A., Molinari N., Pouzeratte Y. (2015). Difficult intubation in obese patients: incidence, risk factors, and complications in the operating theatre and in intensive care units. *British Journal of Anaesthesia*.

[44] De Jong A., Wrigge H., Hedenstierna G. (2020). How to ventilate obese patients in the ICU. *Intensive Care Medicine*.

[45] Schetz M., De Jong A., Deane A. M. (2019). Obesity in the critically ill: a narrative review. *Intensive Care Medicine*.

[46] Bazurro S., Ball L., Pelosi P. (2018). Perioperative management of obese patient. *Current Opinion in Critical Care*.

[47] Ball L., Dameri M., Pelosi P. (2015). Modes of mechanical ventilation for the operating room. *Best Practice & Research Clinical Anaesthesiology*.

[48] Martínez M. F. M. H., Vargas M. A. D. O., Falcón G. C. S., Santos D. G. (2023). Surgical nursing care in the operating room: an integrative review. *Texto Contexto-Enferm*.

[49] Prado P. R. D., Bettencourt A. R. D. C., Lopes J. D. L. (2019). Related factors of the nursing diagnosis ineffective breathing pattern in an intensive care unit. *Revista Latino-Americana de Enfermagem*.

[50] Masoomi H., Blumenauer B. J., Blakkolb C. L., Marques E. S., Greives M. R. (2019). Predictors of blood transfusion in autologous breast reconstruction surgery: a retrospective study using the nationwide inpatient sample database. *Journal of Plastic, Reconstructive & Aesthetic Surgery*.

[51] Ross D., Erkocak O., Rasouli M. R., Parvizi J. (2019). Operative time directly correlates with blood loss and need for blood transfusion in total joint arthroplasty. *The Archives of Bone and Joint Surgery*.

[52] Owens J., Otero J. E., Noiseux N. O., Springer B. D., Martin J. R. (2020). Risk factors for post-operative blood transfusion following total knee arthroplasty. *The Iowa Orthopaedic Journal*.

[53] Lang Ben Nun E., Sela H. Y., Joseph J., Rudelson G., Grisaru-Granovsky S., Rottenstreich M. (2022). Prolonged operative time of cesarean is a risk marker for subsequent cesarean maternal complications. *Archives of Gynecology and Obstetrics*.

[54] Palka J. K., Argade S. P., Gross J. T., Vetter J., Figenshau R. S. (2023). An analysis of post-operative pain and narcotic use following robotic assisted laparoscopic prostatectomy for same day discharge. *Journal of Robotic Surgery*.

[55] Zeeni C., Abou Daher L., Shebbo F. M. (2022). Predictors of postoperative pain, opioid consumption, and functionality after arthroscopic shoulder surgery: a prospective observational study. *Journal of Orthopaedic Surgery*.

[56] Raebel M. A., Newcomer S. R., Bayliss E. A. (2014). Chronic opioid use emerging after bariatric surgery. *Pharmacoepidemiology and Drug Safety*.

[57] Lang F. F., Liu L. Y., Wang S. W. (2023). Predictive modeling of perioperative blood transfusion in lumbar posterior interbody fusion using machine learning. *Frontiers in Physiology*.

[58] Heard J. C., Siegel N., Yalla G. R. (2023). Predictors of blood transfusion in patients undergoing lumbar spinal fusion. *World Neurosurgery*.

[59] Martin B., Mathisen L. (2005). Use of physical restraints in adult critical care: a bicultural study. *American Journal of Critical Care*.

[60] Almomani M. H., Khater W. A., Qasem B. A., Joseph R. A. (2021). Nurses’ knowledge and practices of physical restraints in intensive care units: an observational study. *Nursing Open*.

[61] Lawson T. N., Tan A., Thrane S. E. (2020). Predictors of new-onset physical restraint use in critically ill adults. *American Journal of Critical Care*.

[62] Cui N., Zhang H., Gan S. (2023). Prevalence and influencing factors of physical restraints in Intensive Care Units: a retrospective cohort study. *Risk Management and Healthcare Policy*.

[63] Benbenbishty J., Adam S., Endacott R. (2010). Physical restraint use in intensive care units across Europe: the PRICE study. *Intensive and Critical Care Nursing*.

[64] Chae S. J., Ahn J. H., Kim E. H., Kim H. J. (2012). Nursing action analysis of operation room nurse according to their career ladders. *Journal of Korean Clinical Nursing Research*.

[65] Milone M. T., Hacquebord H., Catalano L. W., Glickel S. Z., Hacquebord J. H. (2020). Preparatory time–related hand surgery operating room inefficiency: a systems analysis. *Hand*.

[66] Badawy M., Espehaug B., Fenstad A. M. (2017). Patient and surgical factors affecting procedure duration and revision risk due to deep infection in primary total knee arthroplasty. *BMC Musculoskeletal Disorders*.

[67] Lee D. J., Ding J., Guzzo T. J. (2019). Improving operating room efficiency. *Current Urology Reports*.

[68] Kassam A. B., Rovin R. A., Walia S. (2017). The operating room of the future versus the future of the operating room. *Otolaryngologic Clinics of North America*.

[69] Russell B. (2022). Understanding the role of the scrub nurse during robotic surgery. *Nursing Standard*.

[70] Lee Y. Y., Park K. O. (2002). Analysis of core interventions of operating room using nursing intervention classification. *Journal of Korean Academy of Nursing Administration*.

[71] Evered L. A., Chan M. T., Han R. (2021). Anaesthetic depth and delirium after major surgery: a randomised clinical trial. *British Journal of Anaesthesia*.

[72] Keller S., Yule S., Smink D. S., Zagarese V., Safford S., Parker S. H. (2020). Episodes of strain experienced in the operating room: impact of the type of surgery, the profession and the phase of the operation. *BMC Surgery*.

[73] Ha R. M., Kwon K. J., Woo J. H., Kim J. A. (2014). Analysis of nursing intensity related to Nursing activities in operating room using the relative value scale for nursing cost. *Journal of Korean Clinical Nursing Research*.

[74] Saxena R. C., Whipple M. E., Neradilek M. B. (2019). Does attending surgeon presence at the preinduction briefing improve operating room efficiency?. *Otolaryngology- Head and Neck Surgery*.

[75] Avery D. M., Matullo K. S. (2014). The efficiency of a dedicated staff on operating room turnover time in hand surgery. *The Journal of Hand Surgery*.

[76] Wolf F. A., Way L. W., Stewart L. (2010). The efficacy of medical team training: improved team performance and decreased operating room delays: a detailed analysis of 4863 cases. *Annals of Surgery*.

[77] Choi J. S., Eun Y. (2006). A study on the clinical competence according to clinical ladder of operating room nurses. *The Journal of Korean Academic Society of Nursing Education*.

